# Development of Cortical Morphology Evaluated with Longitudinal MR Brain Images of Preterm Infants

**DOI:** 10.1371/journal.pone.0131552

**Published:** 2015-07-10

**Authors:** Pim Moeskops, Manon J. N. L. Benders, Karina J. Kersbergen, Floris Groenendaal, Linda S. de Vries, Max A. Viergever, Ivana Išgum

**Affiliations:** 1 Image Sciences Institute, University Medical Center Utrecht, Utrecht, The Netherlands; 2 Department of Neonatology, University Medical Center Utrecht, Utrecht, The Netherlands; 3 Department of Perinatal Imaging & Health, Division of Imaging Sciences & Biomedical Engineering, King’s College London, London, United Kingdom; 4 Brain Center Rudolf Magnus, University Medical Center Utrecht, Utrecht, The Netherlands; Chinese Academy of Sciences, CHINA

## Abstract

**Introduction:**

The cerebral cortex develops rapidly in the last trimester of pregnancy. In preterm infants, brain development is very vulnerable because of their often complicated extra-uterine conditions. The aim of this study was to quantitatively describe cortical development in a cohort of 85 preterm infants with and without brain injury imaged at 30 and 40 weeks postmenstrual age (PMA).

**Methods:**

In the acquired T_2_-weighted MR images, unmyelinated white matter (UWM), cortical grey matter (CoGM), and cerebrospinal fluid in the extracerebral space (CSF) were automatically segmented. Based on these segmentations, cortical descriptors evaluating volume, surface area, thickness, gyrification index, and global mean curvature were computed at both time points, for the whole brain, as well as for the frontal, temporal, parietal, and occipital lobes separately. Additionally, visual scoring of brain abnormality was performed using a conventional scoring system at 40 weeks PMA.

**Results:**

The evaluated descriptors showed larger change in the occipital lobes than in the other lobes. Moreover, the cortical descriptors showed an association with the abnormality scores: gyrification index and global mean curvature decreased, whereas, interestingly, median cortical thickness increased with increasing abnormality score. This was more pronounced at 40 weeks PMA than at 30 weeks PMA, suggesting that the period between 30 and 40 weeks PMA might provide a window of opportunity for intervention to prevent delay in cortical development.

## Introduction

During the third trimester of pregnancy, the cerebral cortex develops from a smooth surface to a folded structure with high complexity, resembling the morphology of the adult cortex [1]. In preterm infants, neurodevelopment is particularly vulnerable due to extra-uterine complications, leading to primary brain injury and secondary developmental consequences [2–4].

In this population, it has been described that the development of the cerebral cortex, with respect to changes in surface area [5,6] and cortical folding [7], might be related to functional and cognitive development. Furthermore, preterm infants exhibit abnormal development of cortical thickness when measured in adolescence [8–10]. In normal development, thinning of the cerebral cortex by pruning of dispensable neurons and synapses, leading to more efficient synaptic connections, occurs during childhood and adolescence [11–14] and continues with aging [15,16]. In preterm infants, this process seems to be disturbed [10]. Some studies found that preterm infants have larger cortical grey matter (CoGM) volumes compared to healthy infants in several regions at term equivalent age [17,18], and in adolescence [19], while others found smaller volumes [20]. Padilla et al. [18] found that preterm infants without focal brain lesions have decreased CoGM volume over the whole brain, but increased grey matter volumes in the occipital lobes.

It has been described that white matter injury (the most common type of brain injury in preterm infants), varying from cystic periventricular leukomalacia, punctate unmyelinated white matter (UWM) lesions, to diffuse UWM injury, may have secondary maturational and trophic consequences due to axonal damage, affecting the microstructural integrity and connectivity between thalamus and cortex [3,4]. This might disturb the early formation, development and maturation of the cerebral cortex.

Magnetic resonance imaging (MRI) is of additional value in indicating infants at the highest risk of neurodevelopmental impairments. Quantitative assessment of cortical development based on MRI, describing volume, cortical surface area, cortical thickness, and cortical folding could potentially aid in evaluating development. These quantitative descriptors and their longitudinal change are interesting when investigated in specific brain regions, in infants with normal development, as well as in cases with brain injury. Altered cortical development of preterm infants has been mainly reported at term equivalent age, childhood, or adolescence [10,18,19,21]. Longitudinal imaging prior to term equivalent age, when most changes take place, is lacking.

The aim of this study is to quantitatively describe morphological changes of the cerebral cortex in a unique cohort of extremely preterm infants with and without brain injury, with clinically acquired longitudinal MR images at 30 and 40 weeks postmenstrual age (PMA).

## Data

This study included 117 longitudinally scanned preterm infants. After exclusion of 17 infants who had at least one image of non-diagnostic quality, e.g. due to movement artefacts, and 15 infants with severe brain injury resulting in inaccurate automatic segmentations, a set of 85 infants, with good quality longitudinal images, remained. These infants had an average gestational age of 26.6 ± 1.0 weeks at birth (range: 24.4─27.9 weeks), were 30.7 ± 0.8 week PMA (range: 28.7─32.7 weeks) at the first scan, and 41.1 ± 0.5 weeks PMA (range: 40.0─42.7 weeks) at the second scan. Imaging was performed in accordance with standard clinical practice at the Neonatal Intensive Care Unit of the Wilhelmina Children’s Hospital of the University Medical Center Utrecht, The Netherlands. Detailed patient information is listed in Table 1. All patients were sedated with oral chloral hydrate (30─60 mg/kg). Coronal T_2_-weighted images were acquired on a Philips Achieva 3T scanner (Philips Medical Systems, Best, The Netherlands) with multislice 2D turbo spin echo sequences, using an echo time of 120 ms at 30 weeks and 150 ms at 40 weeks, and a repetition time of 10085 ms at 30 weeks and 4847 ms at 40 weeks. The images were reconstructed to voxel sizes of 0.34 × 0.34 × 2.0 mm^3^ at 30 weeks PMA and 0.35 × 0.35 × 1.2 mm^3^ at 40 weeks PMA.

**Table 1:** Patient information and the number of patients with brain injury in this cohort.

Number of patients	85
Male/female	42/43
Intraventricular haemorrhage grade I	10 (11.8%)
Intraventricular haemorrhage grade II	13 (15.3%)
Intraventricular haemorrhage grade III	5 (5.9%)
Posthaemorrhagic ventricular dilation	4 (4.7%)
Punctate white matter lesions (> 6)	2 (2.4%)
Cerebellar lesions (> 6) or cerebellar haemorrhage	3 (3.5%)
Lenticulostriate infarction	3 (3.5%)

No patients with cystic periventricular leukomalacia and no patients with periventricular haemorrhagic infarction were found in this cohort.

Permission from the medical ethical review committee of the University Medical Center Utrecht (MERC UMC Utrecht) for the current study and informed parental consent for the MRI was obtained. Patient data were anonymised prior to the analysis. Since this was a retrospective study, using MR images performed as part of standard clinical care, oral consent for the MRI was obtained by the treating physician and any questions or remarks were noted in the charts. No written consent was deemed necessary. The MERC UMC Utrecht waived the need for parental consent for both the use of medical data and for publication of medical images.

All MR images acquired at 40 weeks PMA were scored for brain injury using the assessment tool presented by Kidokoro et al. [2]. This scoring system evaluated unmyelinated white matter abnormality, cortical grey matter abnormality, deep grey matter abnormality, cerebellar abnormality, and total brain abnormality. It included visual assessment as well as manual 2D measurements.

Based on the score, the patients were divided in four classes: normal, mild, moderate, and severe. For total brain abnormality this resulted in: 35 patients in the normal class, 42 in the mild class, 7 in the moderate class, 1 in the severe class. For CoGM abnormality this resulted in: 27 patients in the normal class, 35 in the mild class, 18 in the moderate class, and 5 in the severe class. Because of the small number of patients in the severe classes, the moderate and severe classes were combined in the statistical evaluation.

The interhemispheric fissure width, which was part of the cortical grey matter abnormality score, is recognised as a measure of cerebral atrophy [22] and is therefore also investigated separately.

## Methods

To enable analysis of the cortex, UWM, CoGM, and cerebrospinal fluid in the extracerebral space (CSF) were automatically segmented in the coronal T_2_-weighted images. Subsequently, the brain was divided into frontal, temporal, parietal, and occipital lobes. Finally, the automatic segmentations and parcellations were used to calculate quantitative descriptors characterising cortical development in the whole cohort. A pilot study using these quantitative descriptors has been described by Moeskops et al. [23].

### 3.1. Segmentation and parcellation

**Fig 1:**
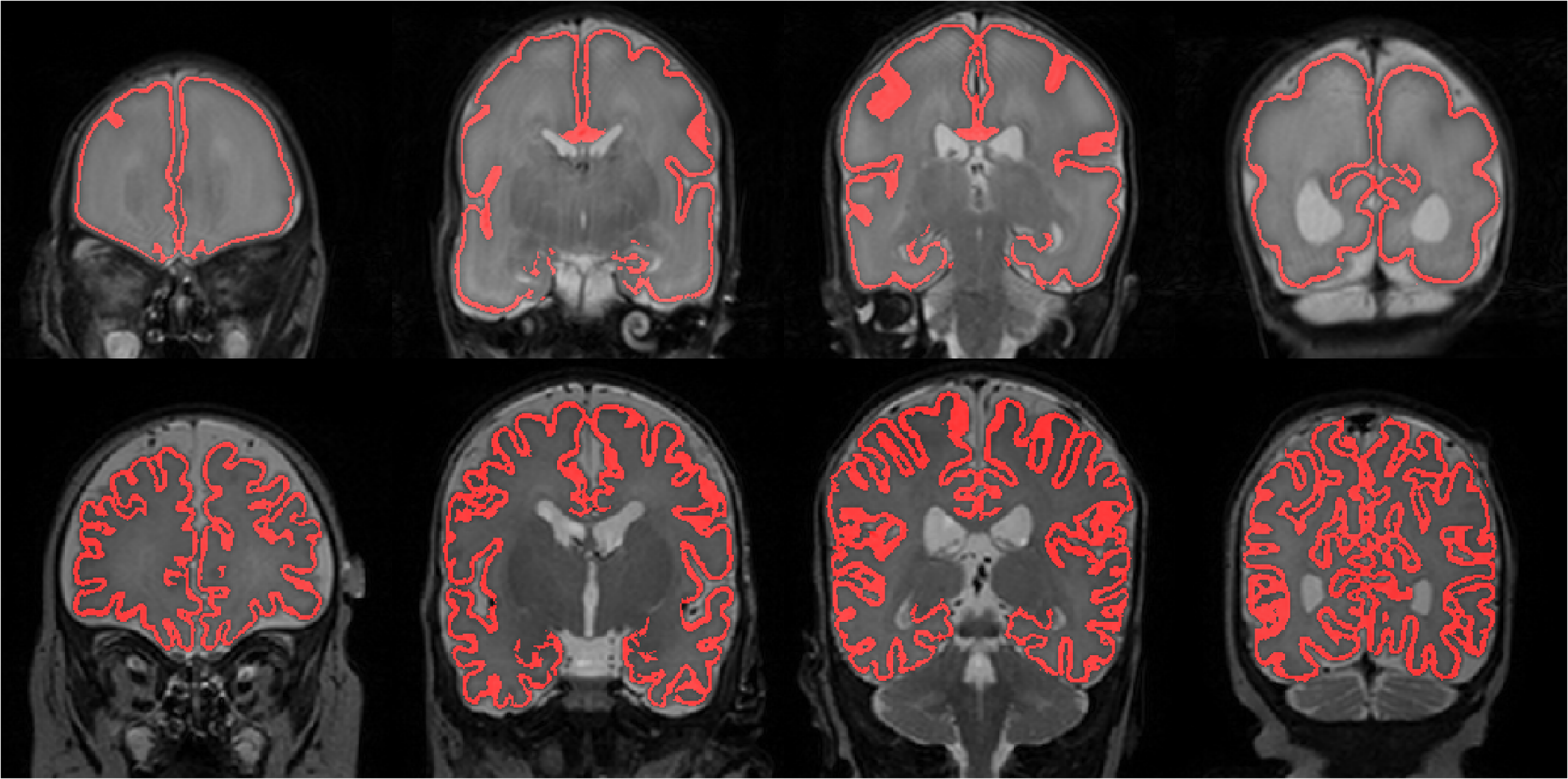
Automatic segmentation of CoGM at 30 (top) and 40 weeks PMA (bottom) for the same patient, shown in four slices of the T2-weighted images. Note that, because of the limited resolution, CSF inside the sulci was not always visible, which resulted in local overestimation of cortical thickness.

UWM, CoGM and CSF were automatically segmented in the coronal T_2_-weighted images using a recently presented supervised classification method [24]. The method has been evaluated in the Neonatal Brain Segmentation challenge (NeoBrainS12), a study comparing the performance of algorithms for automatic neonatal brain segmentation [25]. The results on images acquired with the same protocol as used in this study are available at http://neobrains12.isi.uu.nl/mainResults.php and show better performance of this method compared to other methods. An example of a CoGM segmentation result in images acquired at 30 and 40 weeks PMA of the same patient is illustrated in Fig 1.

**Fig 2:**
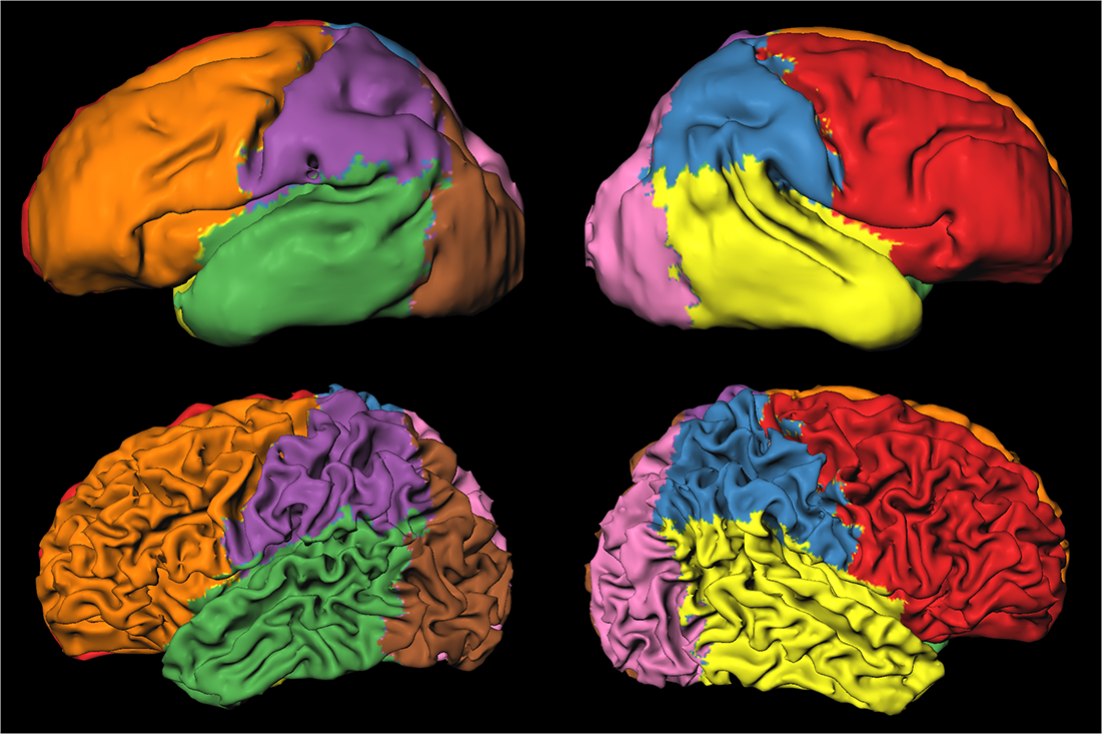
Automatic parcellation of the images acquired at 30 weeks (top) and 40 weeks (bottom) in frontal (red and orange), temporal (yellow and green), parietal (blue and purple), and occipital (pink and brown) lobes. The images were scaled separately and therefore do not show change in size of the brain.

To enable localised analysis, we have automatically parcellated both the right and the left hemisphere of the MR brain images into frontal, temporal, parietal, and occipital lobes, i.e. eight regions, using registration of an annotated template image. An example of a resulting automatic parcellation for the images acquired at 30 and 40 weeks PMA is shown in Fig 2. Details of this procedure can be found in S1 Methods.

### 3.2. Quantification of cortical morphology

**Fig 3:**
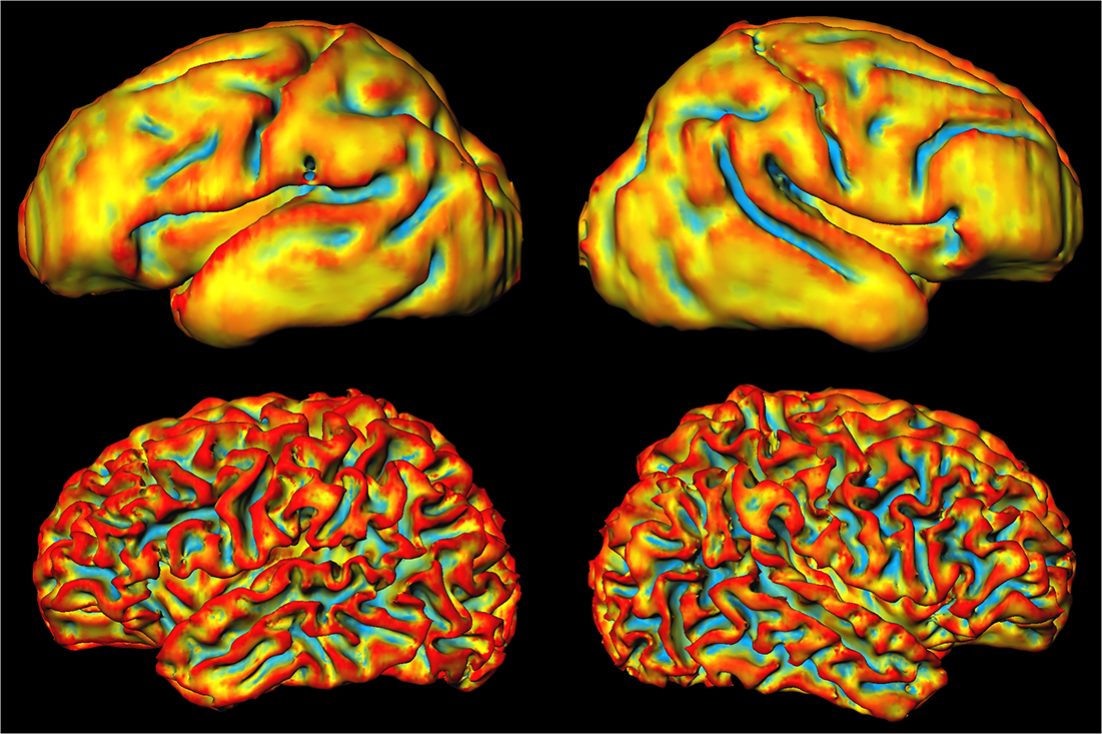
Local mean curvature of the inner cortical surface as obtained by automatic segmentation on the images acquired at 30 weeks PMA (top) and at 40 weeks PMA (bottom) for one patient. Red indicates positive curvature, blue indicates negative curvature, and yellow indicates zero curvature. The images were scaled separately and therefore do not show change in size of the brain.

Based on the automatic segmentation results, several quantitative descriptors were computed. No further topological correction [26] was performed before these computations. UWM, CoGM, and CSF volumes were determined, and the inner and outer CoGM surface areas were estimated using a voxel-based approach that assigns a weight to each voxel on the surface based the configuration of its neighbouring voxels [27]. Furthermore, cortical thickness was computed in 3D following the method described by Jones et al. [28]. Cortical folding was quantified using two different methods: by gyrification index [29] in 3D, and by global mean curvature. The gyrification index was computed as the ratio between the inner CoGM surface and a smooth convex hull segmentation [30] around the UWM segmentation. Global mean curvature was computed by normalised summation [31] of the local mean curvature of the inner and outer CoGM surface combined. An example of the local mean curvature of the inner CoGM surface is shown in Fig 3. Details of these methods can be found in S1 Methods.

These descriptors were computed for the whole brain as well as for each of the brain regions, for the images acquired at 30 weeks PMA as well as for the images acquired at 40 weeks PMA. To evaluate the change between 30 and 40 weeks PMA, longitudinal increase factors were computed for each patient as the ratio between the descriptors at 40 and 30 weeks PMA.

### 3.3. Statistical evaluation

To evaluate whether the descriptors show differences between the brain regions, differences between the abnormality classes, or differences with respect to the development between 30 and 40 weeks PMA, linear mixed modelling was used to estimate these effects for the descriptors computed from the images acquired at 30 and 40 weeks PMA and from their longitudinal increase factors. The statistical evaluation was performed in R (version 3.1.1).

Models were made, per descriptor, for the images acquired at 30 weeks PMA, the images acquired at 40 weeks PMA, and the longitudinal increase factors separately. Categorical variables were used to describe the hemispheres, the lobes and the abnormality scores. To correct for differences in age at the time of scanning, the patient age at the time of the scan was included in the models for the images acquired at 30 weeks, and the time interval between the two scans was included in the models for the increase factors. No influence of age at the time of scanning was found for the images acquired at 40 weeks, so age was not included in those models. Model optimisation was performed based on Akaike’s information criterion [32].

## Results

The results are presented for: the images acquired at 30 weeks PMA, the images acquired at 40 weeks PMA, as well as the longitudinal changes between these two time points. The first subsection of the results presents the global quantitative descriptors evaluated over the whole brain, while the second subsection presents these descriptors evaluated per region. In the third subsection, the descriptors are presented in relation to the abnormality score classes.

### 4.1. Global evaluation

**Fig 4:**
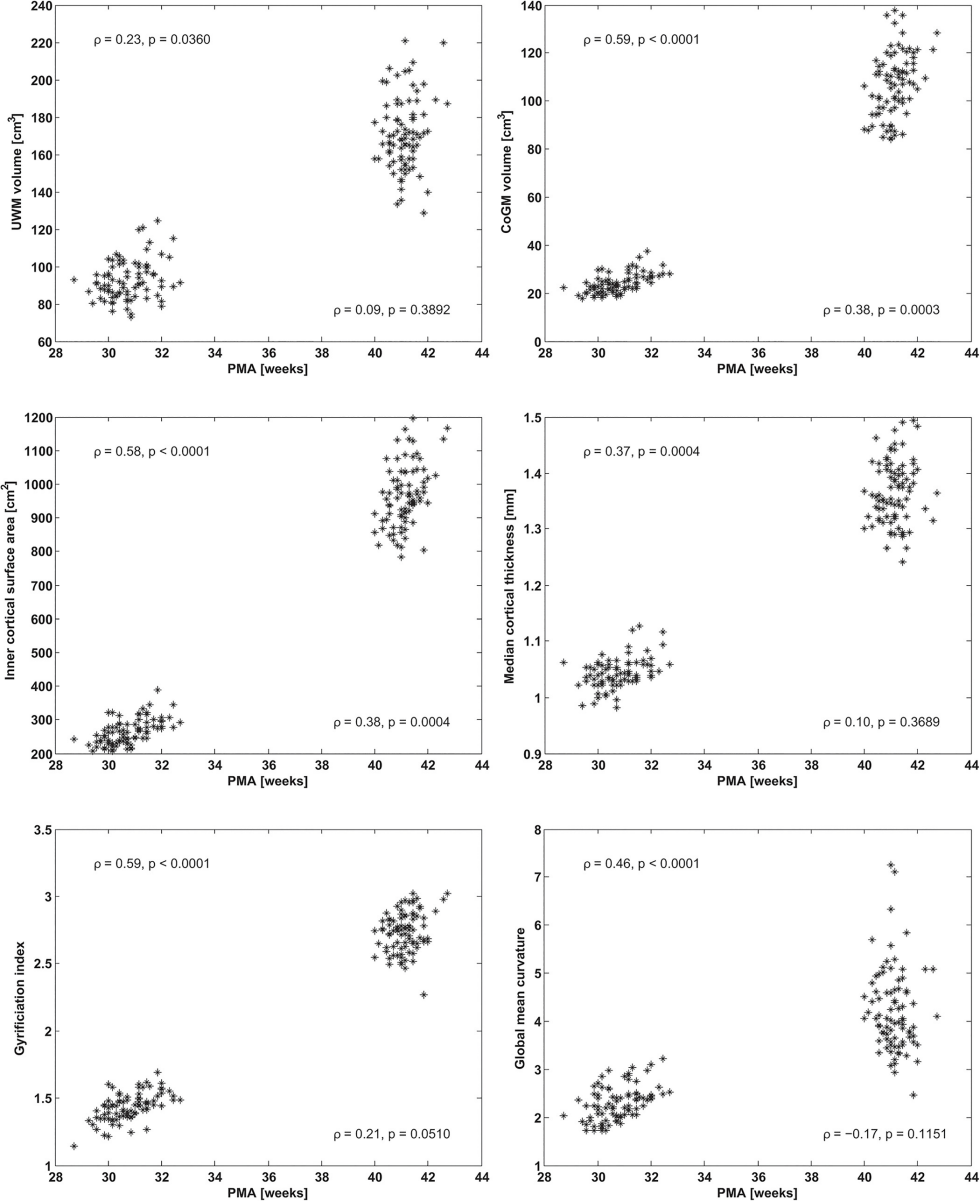
UWM volume (top left), CoGM volume (top right), inner cortical surface area (middle left), median cortical thickness (middle right), gyrification index (bottom left), global mean curvature (bottom right), for the images acquired at 30 and 40 weeks PMA, shown versus PMA at the time of scanning. Spearman’s rank correlation coefficients (*ρ*) and the corresponding *p*-values are shown for 30 (left) and 40 weeks PMA (right) separately.

Fig 4 shows descriptors evaluated over the whole brain versus PMA at the time of scanning, while Table 2 lists the average values and their increase factors. In a period of 10.4 ± 1.0 weeks (mean ± standard deviation) the outer UWM surface area (i.e. inner CoGM surface area) increased by a factor of 3.7, while the UWM volume increased by 1.9. The outer CoGM surface area increased by a factor of 3.3, and the CoGM volume increased by a factor of 4.6. Cortical folding increased by factor 1.9, both in terms of gyrification index and in terms of the global mean curvature of the inner and outer surface. Furthermore, median cortical thickness increased by a factor of 1.3.

**Table 2:** Mean (*μ*) and corresponding standard deviation (*σ*) for the descriptors acquired at 30 and 40 weeks PMA, and their longitudinal increase factors.

Descriptor	30 weeks PMA (*μ* ± *σ*)	40 weeks PMA (*μ* ± *σ*)	Increase factor (*μ* ± *σ*)
UWM volume	93 ± 11 cm^3^	171 ± 19 cm^3^	1.9 ± 0.2
CoGM volume	24 ± 4 cm^3^	107 ± 13 cm^3^	4.6 ± 0.7
CSF volume	53 ± 9 cm^3^	120 ± 22 cm^3^	2.3 ± 0.6
Inner cortical surface area	264 ± 38 cm^2^	964 ± 91 cm^2^	3.7 ± 0.4
Outer cortical surface area	277 ± 39 cm^2^	894 ± 104 cm^2^	3.3 ± 0.5
Median cortical thickness	1.0 ± 0.0 mm	1.4 ± 0.1 mm	1.3 ± 0.1
Gyrification index	1.4 ± 0.1	2.7 ± 0.1	1.9 ± 0.1
Global mean curvature	2.3 ± 0.4	4.2 ± 0.9	1.9 ± 0.5

The computed descriptors were: UWM volume, CoGM volume, CSF volume, inner cortical surface area, outer cortical surface area, median cortical thickness, gyrification index, and global mean curvature of the inner and outer cortical surface together.

### 4.2. Regional evaluation

**Fig 5:**
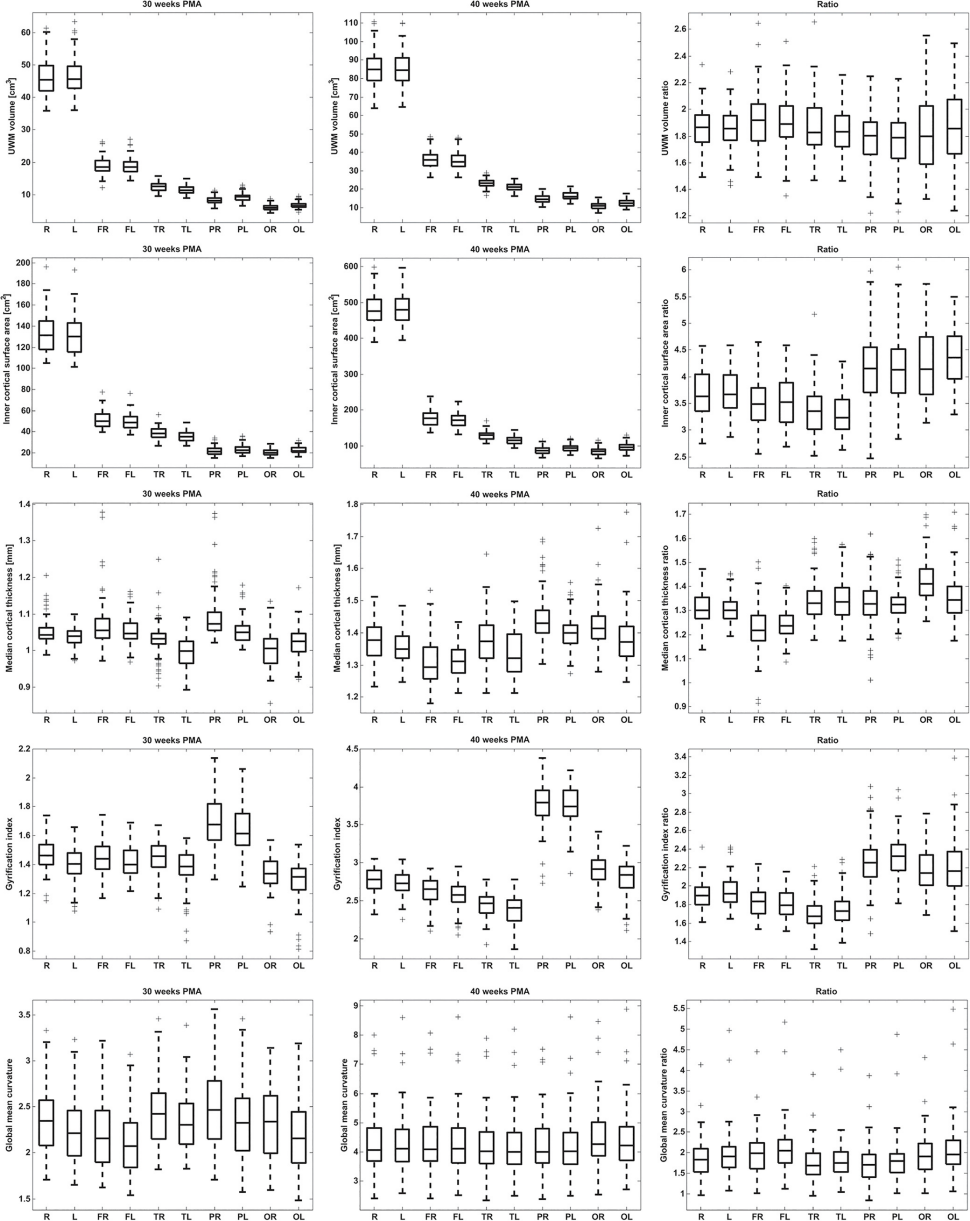
Regional evaluation (in terms of standard boxplots) for, from top to bottom: UWM volume, inner cortical surface area, median cortical thickness, gyrification index, and global mean curvature. The columns show, from left to right: the results for the images acquired at 30 weeks, the results for the images acquired at 40 weeks, and the ratio between the results for the images acquired at 40 and 30 weeks. Note that, for the descriptors acquired at 30 and 40 weeks PMA, UWM volume and inner cortical surface area were dependent of the size of the defined regions of the parcellation, while median cortical thickness, gyrification index, and global mean curvature were independent of size. In every frame the results are shown for the right (R) and left (L) hemispheres, the right (FR) and left (FL) frontal lobes, the right (TR) and left (TL) temporal lobes, the right (PR) and left (PL) parietal lobes, and the right (OR) and left (OL) occipital lobes.

**Fig 6:**
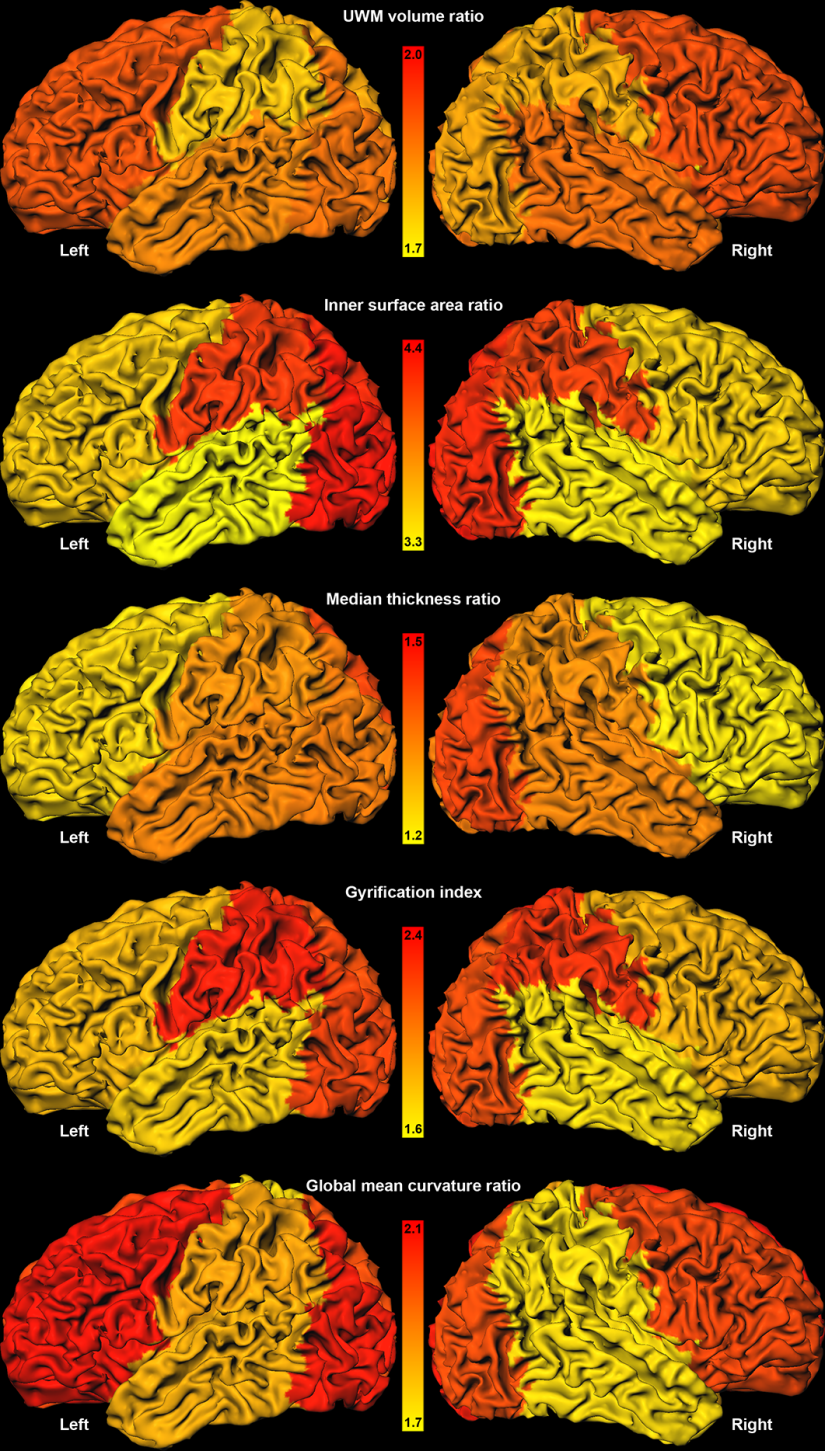
Average regional increase factors for both hemispheres visualised on the inner cortical surface of one randomly chosen patient. The highest increase factor is shown in red and the lowest increase factor is shown in yellow; the range was set separately per descriptor. Note that this figure provides a visualisation of the data in the last column of Fig 5.

Fig 5 shows boxplots for all regional descriptors in ten regions: the right and left hemispheres, as well as the right and left frontal, temporal, parietal, and occipital lobes. The average increase factors between 30 and 40 weeks PMA for these regions are shown in Fig 6. The results of the statistical evaluation obtained with linear mixed modelling can be found in S1, S2 and S3 Tables.

Evaluation of the calculated descriptors per lobe revealed that the brain does not develop equally over all regions. For the images acquired at 30 weeks PMA, lower median cortical thickness, gyrification index, and global mean curvature were found for the occipital lobes than for the whole hemispheres, but these values were higher for the images acquired at 40 weeks PMA. Furthermore, compared to the whole hemispheres, higher median cortical thickness, gyrification index, and global mean curvature were obtained for the parietal lobes at 30 weeks PMA, as well as higher values for median cortical thickness, and gyrification index at 40 weeks PMA. When the increase factors of these descriptors between 30 and 40 weeks PMA were compared (Fig 6), larger increase was found in the occipital lobes for UWM volume, inner cortical surface area, median cortical thickness, gyrification index, and global mean curvature. These findings indicate that more rapid development took place in the occipital part of the brain for all these descriptors between 30 and 40 weeks PMA than over the whole hemispheres. The parietal lobes showed larger increase factors for inner cortical surface area, median cortical thickness, and gyrification index. The temporal lobes only showed larger increase factors for median cortical thickness, and the frontal lobes only for UWM volume and global mean curvature.

### 4.3. Comparison with abnormality scores

**Fig 7:**
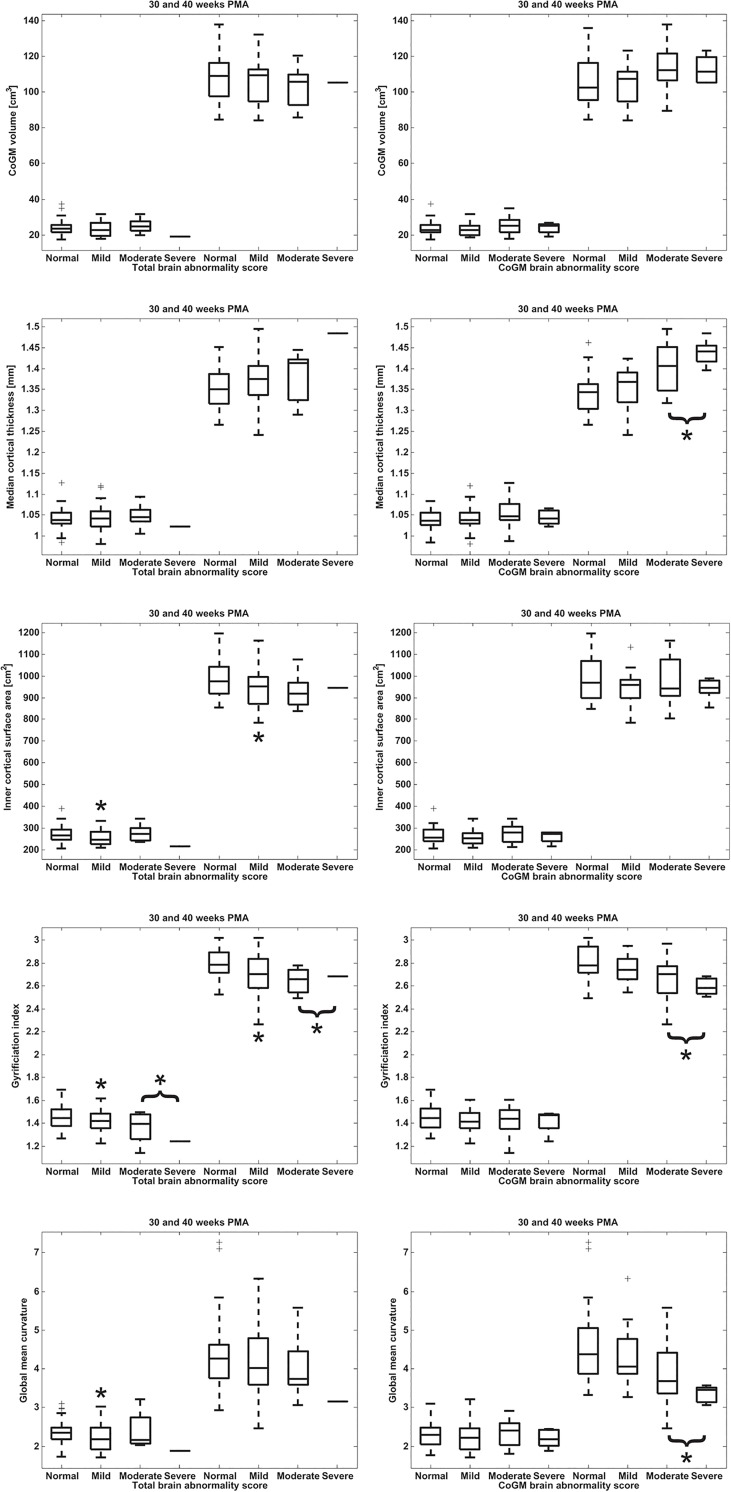
Global cortical morphology descriptors computed from the images acquired at 30 (left) and 40 weeks PMA (right), as a function of total brain (left column) and CoGM abnormality score (right column). From top to bottom: CoGM volume, median cortical thickness, inner cortical surface area, gyrification index, global mean curvature. The four abnormality classes are (i) normal, (ii) mild abnormality, (iii) moderate abnormality, (iv) severe abnormality. Note that there is only one subject in the severe class for the total brain abnormality score. In the statistical evaluation the moderate and severe classes were combined for both the total brain abnormality scoring and the CoGM abnormality scoring. Significant differences with the normal class are indicated with an asterisk (*).

**Fig 8:**
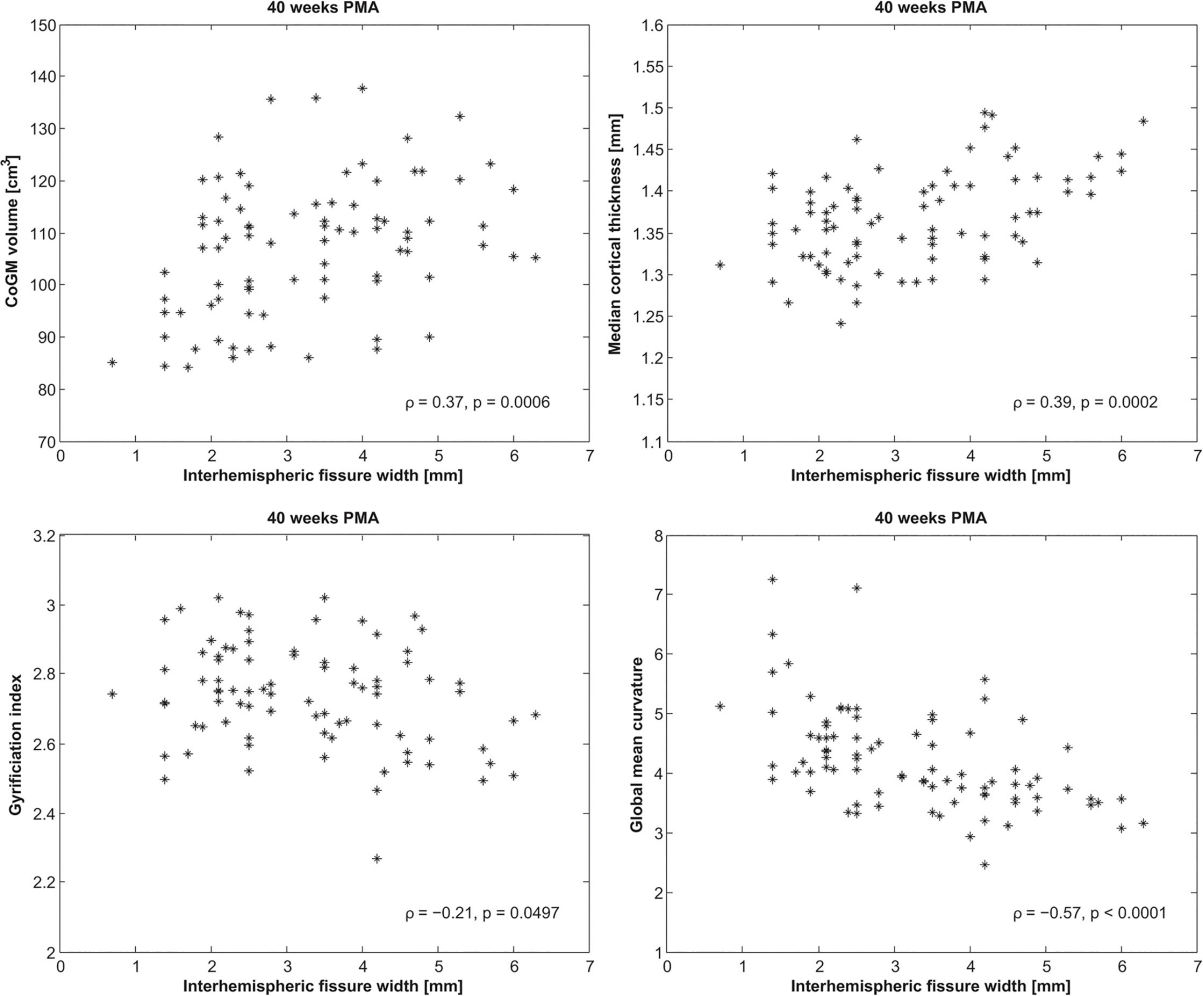
CoGM volume, median cortical thickness, gyrification index, and global mean curvature computed from the images acquired at 40 weeks PMA versus the width of the interhemispheric fissure, measured between the crowns of the superior frontal gyri. Spearman’s rank correlation coefficients (*ρ*) and the corresponding *p*-values are shown at the bottom right. No significant correlation was found for inner cortical surface area.

In Fig 7 the global cortical descriptors computed from the images acquired at 30 and 40 weeks PMA are shown per brain abnormality class. The effects for the patients in these abnormality classes based on linear mixed modelling are listed in Table 3. Fig 8 shows that the cortical descriptors acquired at 40 weeks PMA were significantly correlated with the interhemispheric fissure width, which is part of the CoGM abnornormality scoring and is recognised as a measure of cerebral atrophy [22]. No significant correlation with interhemipheric fissure width was found for the images acquired at 30 weeks PMA.

**Table 3:** Effects of abnormality classes, relative to the normal class, on median cortical thickness, inner cortical surface area, gyrification index, and global mean curvature, computed from the images acquired at 30 and 40 weeks PMA, estimated with linear mixed modelling.

Descriptor	Abnormality class	Effect on descriptor	*p*-value
Median thickness [mm] (40 weeks PMA)	CoGM: mild	+0.0083	0.5096
	CoGM: moderate and severe	+0.067	<0.0001*
Inner cortical surface area [cm^2^] (40 weeks PMA)	Total brain: mild	-8.7	0.0363*
	Total brain: moderate and severe	-11.7	0.0967
Gyrification index (40 weeks PMA)	Total brain: mild	-0.11	0.0011*
	Total brain: moderate and severe	-0.16	0.0075*
	CoGM: mild	-0.07	0.0553
	CoGM: moderate and severe	-0.17	0.0001*
Global mean curvature (40 weeks PMA)	CoGM: mild	-0.29	0.1910
	CoGM: moderate and severe	-0.86	0.0008*
Inner cortical surface area [cm^2^] (30 weeks PMA)	Total brain: mild	-3.7	0.0096*
	Total brain: moderate and severe	-2.0	0.3996
Gyrification index (30 weeks PMA)	Total brain: mild	-0.04	0.0362*
	Total brain: moderate and severe	-0.12	0.0002*
Global mean curvature (30 weeks PMA)	Total brain: mild	-0.15	0.0388*
	Total brain: moderate and severe	-0.06	0.6447

No significant effects of the abnormality score were found for CoGM volume. Significant differences with the normal class are indicated with an asterisk (*), if none of the classes showed a significant effect the descriptor is not shown in this table.

Overall, median cortical thickness increased with increasing abnormality score, while gyrification index and global mean curvature decreased with increasing abnormality score. Using *α* = 0.05, we found a significant increase in median cortical thickness for the images acquired at 40 weeks PMA when comparing the combined moderate and severe CoGM abnormality class with the normal class (no abnormality). For the gyrification index, significant decreases were found when comparing the mild class and the combined moderate and severe abnormality class with the normal class, for total brain abnormality as well as for CoGM abnormality. For global mean curvature, a significant decrease was found when comparing the combined moderate and severe CoGM abnormality class with the normal class. For the images acquired at 30 weeks PMA, significant decreases in gyrification index were found for the mild and the combined moderate and severe total brain abnormality classes compared with the normal class, and a significant decrease in global mean curvature was found for the mild total brain abnormality class compared with the normal class. The same patterns were visible from the longitudinal increase factors between 30 and 40 weeks PMA. No significant influence of UWM abnormality score was found on the descriptors.

## Discussion

This paper evaluated UWM volume, CoGM volume, CSF volume, inner and outer cortical surface area, median cortical thickness, gyrification index, and global mean curvature in preterm infants with longitudinal imaging. These descriptors have been previously related to the state of the brain development [5,6,10,30,31,33–40], but thus far, they have not been investigated in early longitudinal imaging of preterm infants. In agreement with previous findings in cross-sectional studies, this study showed an increase of all evaluated descriptors between 30 and 40 weeks PMA. Significantly more rapid development occurred in the occipital lobes than in other regions. Interestingly, a significant increase in cortical thickness and a significant decrease in cortical folding (in terms of gyrification index and global mean curvature) were found in infants with higher brain abnormality scores according to Kidokoro et al. [2]. These effects were more apparent at 40 weeks PMA than at 30 weeks PMA. The results for gyrification index and global mean curvature indicated agreement between the descriptors that were automatically extracted from the images and visual scoring, as delayed gyrification is included in the CoGM abnormality scoring.

### 5.1. Global brain development

Cross-sectional studies of similar cortical descriptors with preterm neonatal [31,33], as well as foetal MRI [36,41,42], showed an association with age. In our current study, clear increases between 30 and 40 weeks PMA were found for all descriptors (Fig 4). An association with PMA at the time of scanning was found for the images acquired around 30 weeks PMA. This effect was less visible in the images acquired around 40 weeks PMA. This could indicate that the extra-uterine environment, or other consequences of preterm birth, did not yet show an effect on cortical development at 30 weeks PMA, but did have an effect between 30 and 40 weeks PMA, resulting in a larger range of the descriptors at 40 weeks PMA, depending on postnatal complications. Additionally, cortical development occurs less rapidly around 40 weeks PMA than around 30 weeks PMA [36], resulting in a less strong influence of PMA at the time of scanning on the descriptors at 40 weeks PMA than at 30 weeks PMA. No influence of the gestational age at birth was found on the cortical descriptors for the patients in this study.

To compare the difference between development of preterm infants and normal foetal development, analysis of control images of healthy newborns needs to be performed. Unfortunately these data are not available, since no ethical approval has been obtained to scan healthy newborns in our institution so far. Wright et al. [36] recently evaluated different global curvature descriptors applied to foetal MR brain images acquired between 21 and 39 weeks gestational age. With increasing age, similar increases in these descriptors were shown. This study did, however, not evaluate cortical thickness.

### 5.2. Regional brain development

At 40 weeks PMA, the occipital lobes were more mature than the frontal and temporal lobes in terms of gyrification index and global mean curvature. When the increase factors between 30 and 40 weeks PMA were considered, the largest change took place in the occipital lobes. These results are in agreement with the literature suggesting that brain development takes place in an occipital-frontal direction [43–46], which was confirmed by *in vivo* studies [47–50]. The more rapid development in the occipital lobes could explain the earlier functional use and activity of the visual system in preterm infants [18,51,52], in contrast to e.g. behavioural functions involving the frontal lobes. This is supported by previous work suggesting that early neuronal activity of preterm infants relates to subsequent brain growth [53]. Consequently, rapid occipital development could make this region more vulnerable and might be an explanation for the high risk of visual dysfunction in preterm infants [54,55].

The right hemisphere was significantly more mature than the left hemisphere in terms of gyrification index for the images acquired at 30 weeks PMA, as well as the images acquired at 40 weeks PMA. For global mean curvature this was only visible for the images acquired at 30 weeks PMA. This difference between hemispheres corresponds to previous findings in the literature suggesting that the right hemisphere presents gyral complexity earlier than the left hemisphere [33,56].

### 5.3. Cortical development and brain injury

An interesting finding was that patients with a higher (CoGM) abnormality score had increased cortical thickness and decreased cortical folding in terms of gyrification index and global mean curvature. The difference in thickness is significant, however, it is small: on average 1.3 vs. 1.4 mm for the images acquired at 40 weeks PMA, given a voxel size of 0.35 mm. This is a subvoxel difference and therefore difficult to interpret and evaluate visually. Additionally, in this population a positive association was observed between cortical thickness and interhemispheric fissure width measured in 2D (Fig 8), which is part of the CoGM scoring. Cerebral atrophy, expressed as increased hemispheric fissure width, could be a consequence of diffuse UWM injury, with injury to premyelinating oligodendrocytes (preOLs) and a deficit in late-migrating γ-aminobutyric acidergic (GABAergic) neurons. GABAergic neurons are important contributors to the thickness of the upper cortical layers [3,4,45,57,58]. Therefore, disturbed or incomplete migration might appear in the images as a less clear distinction between intensities of UWM and CoGM, which could result in larger cortical thickness estimates. Nosarti et al. [19] investigated tissue volumes of preterm infants measured in adolescence, and suggested that extensive plastic processes might occur to compensate for brain injury, resulting in increases as well as decreases in CoGM volumes in different regions of the brain.

No significant effect of brain abnormality on CoGM volume was found in this study. The opposing relations of brain injury on cortical folding and cortical thickness obtained in this study might be the reason for different findings reported in the literature with respect to CoGM volume in relation to brain abnormalities [17,18,20]. CoGM volume is influenced by cortical surface area, thus by cortical folding, as well as cortical thickness. As suggested by earlier literature and by the results of our study, increase in cortical thickness might indicate disruption of brain development, whereas increase in cortical folding might indicate good development. Therefore, these two counteracting effects on volume could lead either to no visible volume differences, or to differences in either direction, depending on which of the effects is stronger. Hence, CoGM volume alone is likely not a good indicator of cortical development.

The associations of the descriptors with the abnormality scores were less clearly visible for the images acquired at 30 weeks PMA. This corresponds to the previously mentioned larger range of the descriptors acquired at 40 weeks PMA (Fig 4), showing larger deviations in development than when imaged at 30 weeks PMA, likely influenced by the extra-uterine conditions. A critical part of brain development takes place between 30 and 40 weeks PMA and disturbances such as white matter injury can therefore have a large influence on development, i.e. result in secondary brain damage. PreOLs are especially vulnerable to hypoxia-ischemia and inflammation, which are well known processes for white matter injury in preterm infants. As a result of cell death of preOLs, early oligodendrocyte progenitors proliferate and differentiate rapidly, but fail to myelinate normally, ultimately resulting in disrupted development of the cortex as well [59,60]. This damage requires time to develop and become visible and hence the influence on cortical development would be more apparent at 40 weeks PMA. This means that the period between 30 and 40 weeks PMA might offer a window of opportunity for neuroprotective interventions.

No significant influence of UWM abnormality scoring was found on these descriptors. However, interhemispheric fissure width did show a correlation with the cortical descriptors, which might indicate atrophy, possibly due to diffuse UWM injury. Diffuse UWM injury, which is difficult to visualise with conventional imaging, is nevertheless known to influence cortical development [60]. This was further supported by significant differences, in terms of gyrification index and global mean curvature, between normal and mild abnormality scores (Fig 7), indicating that mild injury, possibly involving diffuse UWM injury, might have had an influence on cortical development as well.

The processes behind the development of the cerebral cortex are not yet fully understood. Suggested factors include: genetics [61,62], neuronal differentiation [63–65] and mechanical effects [66–70]. However, in the ferret brain, gyrification seems to arise secondary to cortical processes involving neuronal differentiation [71]. With respect to these mechanical effects, Toro and Burnod [68] have suggested a morphogenetic model of cortical folding in which cortical growth can induce cortical folding by itself. In contrast to this, another theory suggests that specific location and shape of sulci are determined by visco-elastic tensions from white matter tracts connecting cortical regions [67,69,70]. The latter theory might explain why specific abnormalities in the cortical sulcal pattern are observed in certain brain developmental disorders in preterm infants [72–74]. These cortical abnormalities might be a consequence of subtle impairments in neuronal migration and cortico-cortical connections.

In the foetal sheep brain, Rees et al. [75] confirmed delayed gyral formation at mid-gestation caused by sub-acute hypoxemia, an underlying pathophysiological mechanism often described in preterm infants. It is often hypothesised that the disability in preterm infants is primarily associated with impaired neural connectivity with or without tissue loss or visible WMI. Dean et al. [76] found that in the foetal sheep brain reversible cerebral ischemia, even without neuronal loss, showed significant diffuse failure of maturation of cortical pyramidal neurons. This was associated with impaired dendritic growth and synapse formation, consistent with altered connectivity. Delayed decline in cortical fractional anisotropy (FA) on MRI was associated with these changes.

Ball et al. [77] reported that preterm infants at term-equivalent age, in the absence of severe white matter injury, showed a loss of microstructural integrity of the connective white matter tracts. Very preterm infants exhibit cortical neuroplasticity due to reduced thalamo-cortical connectivity compared with term-born controls. Clinical and experimental studies are showing that early WMI is associated with a reduction in cortical complexity. It seems that grey matter damage due to loss of connections, rather than cell loss, plays a major factor in long-term disability [78]. Whether disturbances in connectivity, without visible UMW abnormalities, are related with developmental delay of cortical folding is unclear. Longitudinal MRI studies quantifying cortical development and changes in connectivity with long-term follow-up will provide more insight.

### 5.4. Limitations

The presented study has several limitations. First, the resolution of the acquired images is limited, which resulted in regions where the CSF inside the sulci was not always visible. Therefore, the outer cortical surface, i.e. the surface between CoGM and CSF, was not always well recognised in these regions. This can also be seen from the average outer cortical surface area computed at 40 weeks PMA (Table 2), which is smaller than the average inner cortical surface area. Cortical thickness in these regions was therefore sometimes overestimated as well. To reduce the influence of this effect on the global descriptors for cortical thickness, median values have been presented. In addition to this, cortical thickness is difficult to estimate based on MRI because of the ongoing development of the cortex [79]. Second, the evaluated descriptors were computed fully automatically from the images. This allowed analysis of a large set of images, but could generate errors human observers do not make. However, the performance of the applied segmentation method was similar to inter-observer variability as shown in the NeoBrainS12 study [25], which suggests that these errors were minimal, and therefore not likely to have had a large influence on the computed descriptors. Third, it should be noted that the descriptors used in this study cannot be directly compared across studies, because they are influenced by several factors such as the acquisition protocol, the segmentation method, the definition of the tissue types, the definition of the descriptors, and details of the implementation. A wide range of values was reported in studies that included patients within the same age range (including studies with foetal imaging and term-born infants). For inner CoGM surface area, the values range from 150 to 1500 cm^2^ [30,31,33,34,37,80]. In our study the average surface area increases from 264 to 964 cm^2^ (Table 2). The computation of the cortical surface area is highly dependent on the scale of measurement (this is commonly referred to as the *coastline paradox*), which could, in addition to the above mentioned factors, explain the large range of values in the literature. For cortical thickness, values between 1.2 to 1.4 mm were found by Xue et al. [80]. In our study the average cortical thickness increased from 1.0 to 1.4 mm (Table 2). For gyrification index, values between 1.1 and 2.2 were reported [31,40]. In our study the average gyrification index increased from 1.4 to 2.7 (Table 2), which is larger than found in the mentioned studies. These studies also report a lower cortical surface area, which could suggest that they are based on a smoother definition of the cortical surface, which directly influences the gyrification index as well. For global mean curvature, values from 2 to 10 were reported [31,36], based on several definitions. In our study the average global mean curvature increased from 2.3 to 4.2 (Table 2). Fourth, the number of patients in the severe abnormality class was small. Therefore, statistical conclusions were drawn for patients in the combined moderate and severe class. Finally, our work analysed longitudinally acquired images at fixed time points, while other studies [31,33,36] focused on single images of patients acquired cross-sectionally at different ages. Our study did not allow the analysis of the development over the full age range, but the strength of this approach was that it did allow us to evaluate the (regional) change of the quantitative descriptors within a patient.

## Conclusion

The presented study, automatically quantifying cortical development, provides insight in regional and longitudinal development based on T_2_-weighted MR brain images of preterm infants with and without brain injury, longitudinally acquired at 30 and 40 weeks PMA. The evaluated descriptors showed a larger change in the occipital lobes than in the other lobes. Interestingly, increased cortical thickness and decreased cortical folding were found in infants with brain injury scored according to a conventional abnormality scoring system. This was more pronounced at 40 weeks PMA than at 30 weeks PMA, suggesting that the cortical maturation processes during these 10 weeks might be affected by brain injury or other consequences of preterm birth. This might offer a window of opportunity for neuroprotective intervention. The potential of these findings to predict long-term cognitive outcome and the effect of specific clinical risk factors should be evaluated in further studies.

## Supporting Information

S1 Methods Additional information about the methods. (DOC)

S1 Table Regional effects for the images acquired at 30 weeks PMA, estimated with linear mixed modelling. The effects are relative to the estimated value over the left hemisphere. If there was an additional right-left difference for a specific lobe, this was modelled as an interaction. Note that UWM volume and inner cortical surface area were dependent of the size of the defined regions of the parcellation, while median cortical thickness, gyrification index, and global mean curvature were independent of size. The age at the time of scanning, as well as the abnormality score were included in the models.
(DOC)

S2 Table Regional effects for the images acquired at 40 weeks PMA, estimated with linear mixed modelling. The effects are relative to the estimated value over the left hemisphere. If there was an additional right-left difference for a specific lobe, this was modelled as an interaction. Note that UWM volume and inner cortical surface area were dependent of the size of the defined regions of the parcellation, while median cortical thickness, gyrification index, and global mean curvature were independent of size. The abnormality score was included in the models.
(DOC)

S3 Table Regional effects for the longitudinal increase factor between the images acquired at 30 and 40 weeks PMA, estimated with linear mixed modelling. The effects are relative to the estimated value over the left hemisphere. If there was an additional right-left difference for a specific lobe, this was modelled as an interaction. Note that UWM volume and inner cortical surface area were dependent of the size of the defined regions of the parcellation, while median cortical thickness, gyrification index, and global mean curvature were independent of size. The time interval between the scans was included in the models.
(DOC)
