# Supplementary material for: Development of Cortical Morphology Evaluated with Longitudinal MR Brain Images of Preterm Infants
Source: PLoS One. 2015 Jul 10;10(7):e0131552. doi: 10.1371/journal.pone.0131552 (PMC4498793; doi:10.1371/journal.pone.0131552)
Supplement: S1 Methods — (DOC) [file pone.0131552.s001.doc]

# S1 Methods

## Lobular parcellation

The images were parcellated into frontal, temporal, parietal and occipital lobes using an atlas registration method. For this purpose, a template image was first generated based on nine images acquired at 30 weeks PMA from the same cohort. The template was constructed by iterative, nonlinear co-registration and averaging [1]. For this purpose, rigid, affine, and elastic registrations were performed, using adaptive stochastic gradient descent as optimiser, mutual information as metric, and a multi-resolution approach. Registrations were performed using elastix [2]. The generated template was manually divided into the eight aforementioned regions. To propagate this parcellation to all images used in this study, the template image was first registered to each image acquired at 30 weeks PMA and subsequently propagated within each patient to the image acquired at 40 weeks PMA, using the registration parameters described above.

## Resizing to isotropic voxels.

Prior to the analysis, the images, as well as the segmentations and parcellations were resized to isotropic voxels. For both sets of images, the in-plane voxel spacing (*dv*) was small compared with the slice thickness (*ds*). This sometimes resulted in an abrupt change in anatomy between neighbouring slices, which could result in unreliable cerebral descriptors. To minimise this effect, and to obtain a smooth cortical surface, all images were resized to isotropic voxels. As a result of this resizing, the isotropic images contained new, interpolated slices, where the automatic segmentation was lacking. The segmentation was performed on the original anisotropic images and therefore only slices in the isotropic images that were also present in the original images contained a segmentation. This means that one in *ds*/*dv* slices of the isotropic image had a corresponding segmentation. To acquire a segmentation in the remaining slices of the isotropic image, the slices with a corresponding segmentation were sequentially non-linearly registered to the nearest slices without a segmentation, using normalised mutual information as metric and a gradient descent optimiser. The corresponding segmentations and parcellations were transformed accordingly. This resulted in voxel sizes of 0.34 × 0.34 × 0.34 mm3 for the images acquired at 30 weeks PMA and 0.35 × 0.35 × 0.35 mm3 for the images acquired at 40 weeks PMA, with corresponding segmentations and parcellations.

## Cortical thickness

Cortical thickness was quantified following the method described by Jones et al. [3]. First, a potential field between the inner and outer cortical surfaces was generated from the segmentation of CoGM. Local thickness, for each voxel in the CoGM segmentation, was defined as the length of the curve perpendicular to every isosurface within this potential field, and was calculated by integrating the vector field describing the gradient directions of the potential field between the inner and outer cortical surfaces.

## Cortical curvature

Local curvature of the CoGM surface can be characterised by the principal curvatures $k_{1}$and $k_{2}$($k_{1}>k_{2}$). From $k_{1}$and$k_{2}$, the mean curvature, $H=\frac{1}{2}(k_{1}+k_{2})$ was calculated as a shape descriptor. An example of local mean curvature computed from the automatic segmentation results for one patient is shown in Error: Reference source not found. Based on these local measurements, global descriptors ($H_{\bar{H}}$) were calculated by normalisation [4] over all voxels on the CoGM surface ($S$) using the average mean curvature ($\bar{H}$):

$$H_{\bar{H}}=\frac{1}{\left|\bar{H}\right|}\sqrt{\frac{1}{\sum_{k\in S} a_{k}}\sum_{k\in S} a_{k}H_{k}^{2}}$$

where: $\bar{H}=\frac{1}{\sum_{k\in S} a_{k}}\sum_{k\in S} a_{k}H_{k}$, calculated over the whole brain, or over the hemisphere containing the region at hand, and where$a_{k}$is the local cortical surface area and $H_{k}$is the local mean curvature represented by voxel $k$. When evaluated over both the voxels on the inner ($S_{i}$)*­*  and outer surface ($S_{o}$) the combined value ($H_{\bar{H}_{io}}$) was calculated using:

$$H_{\bar{H}_{io}}=\frac{1}{\left|\bar{H}_{io}\right|}\sqrt{\frac{1}{\sum_{k\in S_{i}} a_{k}}\sum_{k\in S_{i}} a_{k}H_{k}^{2}+\frac{1}{\sum_{k\in S_{o}} a_{k}}\sum_{k\in S_{o}} a_{k}H_{k}^{2}}$$

where: $\bar{H}_{io}=\frac{1}{\sum_{k\in S_{i}} a_{k}}\sum_{k\in S_{i}} a_{k}H_{k}+\frac{1}{\sum_{k\in S_{o}} a_{k}}\sum_{k\in S_{o}} a_{k}H_{k}$.

# References

1. Jongen C, Pluim JPW, Nederkoorn PJ, Viergever M a, Niessen WJ. Construction and evaluation of an average CT brain image for inter-subject registration. Comput Biol Med. 2004;34: 647–62. doi:10.1016/j.compbiomed.2003.10.003

2. Klein S, Staring M, Murphy K, Viergever MA, Pluim JPW. Elastix: a Toolbox for Intensity-Based Medical Image Registration. IEEE Trans Med Imaging. 2010;29: 196–205. doi:10.1109/TMI.2009.2035616

3. Jones SE, Buchbinder BR, Aharon I. Three-dimensional mapping of cortical thickness using Laplace’s Equation. Hum Brain Mapp. 2000;11: 12–32. Available: http://onlinelibrary.wiley.com/doi/10.1002/1097-0193(200009)11:1<12::AID-HBM20>3.0.CO;2-K/full

4. Rodriguez-Carranza CE, Mukherjee P, Vigneron D, Barkovich J, Studholme C. A framework for in vivo quantification of regional brain folding in premature neonates. Neuroimage. 2008;41: 462–478. doi:10.1016/j.neuroimage.2008.01.008
