# Supplementary material for: Development of Cortical Morphology Evaluated with Longitudinal MR Brain Images of Preterm Infants
Source: PLoS One. 2015 Jul 10;10(7):e0131552. doi: 10.1371/journal.pone.0131552 (PMC4498793; doi:10.1371/journal.pone.0131552)
Supplement: S1 Table — The effects are relative to the estimated value over the left hemisphere. If there was an additional right-left difference for a specific lobe, this was modelled as an interaction. Note that UWM volume and inner cortical surface area were dependent of the size of the defined regions of the parcellation, while median cortical thickness, gyrification index, and global mean curvature were independent of size. The age at the time of scanning, as well as the abnormality score were included in the models. (DOC) [file pone.0131552.s002.doc]

S1 Table: Regional effects for the images acquired at 30 weeks PMA, estimated with linear mixed modelling. The effects are relative to the estimated value over the left hemisphere. If there was an additional right-left difference for a specific lobe, this was modelled as an interaction. Note that UWM volume and inner cortical surface area were dependent of the size of the defined regions of the parcellation, while median cortical thickness, gyrification index, and global mean curvature were independent of size. The age at the time of scanning, as well as the abnormality score were included in the models.

| **Descriptor** | **Region** | **Effect on descriptor** | ***p*-value** |
| --- | --- | --- | --- |
| UWM volume [cm^3^] | Right hemisphere | -0.7 | 0.0001 |
|  | Frontal lobes | -27.7 | <0.0001 |
|  | Temporal lobes | -34.9 | <0.0001 |
|  | Parietal lobes | -37.4 | <0.0001 |
|  | Occipital lobes | -39.8 | <0.0001 |
|  | Right frontal lobe | +0.9 | 0.0088 |
|  | Right temporal lobe | +1.6 | <0.0001 |
| Inner cortical surface area [cm^2^] | Right hemisphere | +1.9 | 0.0008 |
|  | Frontal lobes | -81.1 | <0.0001 |
|  | Temporal lobes | -94.8 | <0.0001 |
|  | Parietal lobes | -107.9 | <0.0001 |
|  | Occipital lobes | -108.3 | <0.0001 |
|  | Right parietal lobe | -3.4 | 0.0031 |
|  | Right occipital lobe | -4.0 | 0.0004 |
| Median cortical thickness [mm] | Right hemisphere | +0.016 | <0.0001 |
|  | Frontal lobes | +0.021 | <0.0001 |
|  | Temporal lobes | -0.038 | <0.0001 |
|  | Parietal lobes | +0.019 | 0.0001 |
|  | Occipital lobes | -0.015 | 0.0018 |
|  | Right temporal lobe | +0.014 | 0.0215 |
|  | Right parietal lobe | +0.022 | 0.0005 |
|  | Right occipital lobe | -0.039 | <0.0001 |
| Gyrification index | Right hemisphere | +0.06 | <0.0001 |
|  | Temporal lobes | -0.02 | 0.0066 |
|  | Parietal lobes | +0.22 | <0.0001 |
|  | Occipital lobes | -0.12 | <0.0001 |
| Global mean curvature | Right hemisphere | +0.12 | <0.0001 |
|  | Frontal lobes | -0.13 | <0.0001 |
|  | Temporal lobes | +0.10 | <0.0001 |
|  | Parietal lobes | +0.12 | <0.0001 |
|  | Occipital lobes | -0.03 | 0.0126 |
